# Why applied science is ignored: and why that matters

**DOI:** 10.1177/14034948251381532

**Published:** 2025-10-16

**Authors:** Töres Theorell

After a long career in internal medicine, social medicine and stress research with components of physiology, epidemiology and intervention trials beginning in the 1960s, I have been struck during later years by the increased lack of interest in results from applied research in societal decision making. In the 1980s, research with implications for society seemed to be in higher regard than it is at present [1, 2]. At present, the trust among lay people in applied research is low, particularly in social and humanistic research. According to repeated population surveys performed by the SOM institute in Göteborg from 2002 to 2020, trust in results from societal research in the general population has been around 50% with small variations. At the same time the trust in medical research has been better, between 80% and 70% with a slight downward trend from 2010 during that period [3]. My understanding is that this important phenomenon has not been discussed very much in the scientific community. In my book ‘Underuse of applied science’ [4], I try to discuss the problem from many points of view. Underuse is discussed both from external points of view (trust among lay people, attitudes among politicians) and from the inside (recruitment of researchers, research education, violations of scientific rules, financial processes). My own basis has been Sweden, a small European country with old and strong democratic and scientific traditions. Tendencies that I describe are similar in other western European countries.

The book has the following parts:

1. Production and producers of applied science
2. Research students
3. How does applied science operate?
4. Communication with society
5. Examples of situation in which applied should have been used more
6. Solutions
7. Conclusions

During my own researcher period, lasting from the 1960s to the 2020s, many profound changes in the world of research have taken place. For instance, the number of doctoral dissertations has increased substantially. At the Karolinska Institutet in Stockholm, the number has increased from approximately 100 per year in the 1970s to around 350 in the 2020s. This is an international trend at universities. At the same time there has been an increase in the number of scientific reports and journals. This is a good thing in itself, but it also creates problems. It has become more and more difficult for politicians to make the right choices in the large amounts of diverse opinions among researchers. Accordingly, reliable research may be dismissed for the wrong reasons. And conversely, not so well founded research is accepted also for the wrong (but other) reasons.

Several problems arise in the large production of research, particularly in journals that depend on researchers who need to get their research published. This gives rise to a growing problem, which is mirrored in the fact that some journals and publishers are labelled ‘predatory’ There are lists of predatory journals [5], which researchers are recommended to avoid publishing in. The term predator refers to a non-critical attitude to scientific production. Their slogan could be ‘we publish anything regardless of scientific quality if you pay’ – of course no journal or publisher would use such a slogan, but those on the list are suspected of using this principle in their decisions to publish.

Some politicians stick to erroneous ideas which their electorates like and which it is almost impossible for those politicians to back away from. They fear loss of voters, and as they may not be well educated in research they may find published research results that are not accepted in well founded science but happen to fit the political ideas they want to embrace (see for instance Cleetus, 2025 [6]). Commercial forces may stimulate the production of faked results that favour their own production and inhibit tyhe publication of more reliable results that speak against.

One common publication problem is ‘positive publication bias’. This means that a journal may be more willing to publish results verifying rather than refuting a hypothesis, even when results are not convincing. In the book, a graphic example presents a method for revealing such a tendency in surveys of a research field [7].

With regard to solutions, underuse of applied research arises on many levels in society. It is impossible to blame one group exclusively. We have to work with all parts of the system. And we as scientists have to explore our own contribution to underuse of our results and not only blame all the others. First of all, researchers have the ethical obligation to follow the rules that they have formulated themselves. The researcher community also has to spread knowledge in the general population and among journalists about research principles. For instance, informed journalists and readers should know about concepts such as positive bias, lack of statistical power and low participation rate. This would enable critical reading of scientific reports among lay people. In consequence of this we need to introduce the subject ‘research knowledge’ in our high schools. Such programmes are being tested [8, 9].

It is my hope that this book will contribute to a constructive debate on how to improve the situation.
